# Association Study of IGF-1 rs35767 and rs6214 Gene Polymorphisms with Cancer Susceptibility and Circulating Levels of IGF-1, IGFBP-2, and IGFBP-3 in Colorectal Cancer Patients

**DOI:** 10.3390/biomedicines11123166

**Published:** 2023-11-28

**Authors:** Maryam H. Alrashid, Ahmad E. Al-Serri, Rubina F. Hussain, Suzanne A. Al-Bustan, Jasem Al-Barrak

**Affiliations:** 1 Department of Biological Sciences, Faculty of Science, Kuwait University, Safat, Kuwait City 13060, Kuwait; rubina.hussain@ku.edu.kw (R.F.H.); s.albustan@ku.edu.kw (S.A.A.-B.); 2 Human Genetics Unit, Department of Pathology, Faculty of Medicine, Kuwait University, Safat, Kuwait City 13060, Kuwait; ahmad.alserri@ku.edu.kw; 3 Kuwait Cancer Control Center, Kuwait City 70030, Kuwait; jaalbarrak@moh.gov.kw

**Keywords:** insulin-like growth factor-1, colorectal cancer, biomarkers, gene polymorphism

## Abstract

Early detection of colorectal cancer (CRC) increases the 5-year survival rate by 90%; therefore, non-invasive biomarkers such as measurable circulating proteins for early detection and prognosis are crucial. Insulin-like growth factor-1 (IGF-1) is involved in the regulation of cell proliferation and apoptosis. IGF binding proteins (IGFBPs) bind and inhibit the activity of IGF-1. It was inconsistently reported that high IGF-1 and IGFBP-2 and low IGFBP-3 circulating levels are associated with high cancer risk, poor prognosis, and tumor metastasis in several cancers. A total of 175 patients with CRC and 429 controls were enrolled in this study. We genotyped for IGF-1 rs35767 and rs6214 gene polymorphisms and assessed their association with circulating levels of IGF-1 and/or the risk for CRC. We also determined plasma levels of IGF-1, IGFBP-2, and IGFBP-3. Neither rs35767 nor rs2614 were associated with cancer risk or IGF-1 levels in our study cohort. IGF-1 and IGFBP-3 levels were higher in controls than in patients, whereas IGFBP-2 was higher in patients than in controls. Only IGFBP-2 was associated with increased tumor grade but not stage. Therefore, IGF-1, IGFBP-2, and IGFBP-3 may be useful as early detection and prognostic biomarkers in CRC.

## 1. Introduction

Colorectal cancer (CRC) is the fourth most common and most deadly cancer in the world [1]. Detecting CRC at a localized stage increases the 5-year survival rate by up to 90%. Therefore, the search for non-invasive and consistent biomarkers such as measurable circulating proteins is of critical importance. The insulin-like growth factor-1 (IGF-1) signaling pathway is involved in the regulation of cell proliferation, survival, differentiation, and apoptosis [2]. The main constituents of the IGF-1 signaling pathway are the circulating growth factor IGF-1, cell surface receptor IGF receptor 1 (IGF-1R), and IGF binding proteins 1–6 (IGFBP 1–6). IGF-1R is a receptor tyrosine kinase, and its activation stimulates coordinated cell growth by activating downstream mitogenic proteins via the Ras pathway and survival proteins such as AKT [3,4]. This ultimately leads to the activation of cell cycle activators such as Cyclin-D and cyclin-dependent kinase 4/6 (CDK4/6) and the inhibition of cell cycle suppressors such as KIP proteins and phosphatase and tensin homologue [5].

IGFBPs bind to and inhibit the activity of IGF-1. Thus, IGF-1 exerts growth-stimulatory effects, whereas IGFBPs exert growth-inhibitory effects. In healthy adults, IGF-1 is bound to one of the IGFBPs, primarily IGFBP-3, and to a lesser extent, IGFBP-2, in circulation. [2,6]. Studies have demonstrated that low IGFBP-3 and high IGF-1 and IGFBP-2 levels are associated with high cancer risk, poor prognosis, and tumor metastasis in several cancers, including CRC, breast cancer, and prostate cancer; however, these findings remain controversial and can be population specific [7,8,9].

Many studies reported that high levels of circulating IGF-1 are associated with CRC; however, other studies showed contradictory results. For instance, a study on approximately 400,000 individuals from the UK biobank showed a positive correlation between circulating IGF-1 and CRC, while another from the European Prospective Investigation into Cancer and Nutrition (EPIC) cohort reported no association [10,11].

In addition to inhibiting IGF-1, IGFBP-2 and IGFBP-3 have been both shown to have IGF-1-indepenent effects [12]. IGFBP-2 is involved in pro-oncogenic functions such as increasing cell migration, invasion, and angiogenesis through nuclear and intercellular mechanisms that lead to transcriptional activation of vascular endothelial growth factor (VEGF) [13,14,15,16]. Increased circulating levels of IGFBP-2 have been detected in both the serum and tumor tissues of most cancers including CRC, and this has been shown to be associated with worse prognosis [17,18,19,20].

On the other hand, IGFBP-3, the most abundant IGFBP in circulation, is involved in anti-tumor functions such as inhibition of cell proliferation and induction of apoptosis and cell cycle arrest [21,22,23]. IGFBP-3 is upregulated at the transcriptional level by p53, and this activation is necessary for p53-induced apoptosis, and hence, the prevention of abnormal cell growth [24]. p53, possibly the most important human tumor-suppressor gene, is activated in response to DNA damage to induce DNA repair, cell cycle arrest, or apoptosis and is mutated in 50–60% of all human cancers [25,26,27]. This indicates that IGFBP-3 works in a tumor-suppression mode; therefore, it would be logical for it to be downregulated in cancer. Studies show major discrepancies in this regard, while the majority of studies show lower circulating levels of IGFBP-3 in several cancers including hepatocellular carcinoma [28], esophageal cancer [7], and CRC [29]. However, other studies show contradictory results where no association between IGFBP-3 and CRC is found [9].

Single nucleotide polymorphisms (SNPs) are the most common genetic variations among individuals. Numerous SNPs have been shown to be associated with clinical characteristics such as cancer risk [30]. Therefore, SNPs can be used as biomarkers for risk assessment and cancer screening; however, many SNPs show a population-specific association [31]. Thus, it is necessary to investigate if an SNP can be used as a universal or population-specific biomarker. Single SNPs in IGF-1 have been associated with increased circulating levels of IGF-1 and/or cancer risk [32,33]. The most significant SNP that exhibits an association with increased levels of circulating IGF-1 and is also associated with an increased risk of CRC is IGF-1 rs35767 (A > G) [32]. Although this risk has been reported in several populations, a few studies showed that this is not the case in all populations. For instance, a multiethnic study in the American population showed that rs35767 was associated with a lower risk for CRC in Latinos and a null association for other racial/ethnic groups in the study [34]. Another polymorphism of interest is IGF-1 rs6214 (C > T), which was observed to be associated with an increased risk for CRC in the Austrian population but not in the Iranian population [35,36]. Taken together, these results necessitate the investigation of the effect of IGF-1 SNPs in different populations to establish them as CRC risk indicators.

In this study, we aimed to determine the circulating levels of IGF-1, IGFBP-2, and IGFBP-3 in patients with CRC and to investigate their association with different clinical aspects of CRC, including risk, tumor grade, and tumor stage. We also assessed the genotypic and allelic frequencies of SNPs rs35767 and rs6214, their association with plasma IGF-1 levels, and the risk for CRC.

## 2. Materials and Methods

### 2.1. Characteristics of Study Cohort

Blood samples, relevant biostatistics, and background data were collected from volunteers aged 18–75 years of both sexes. The controls were individuals without a history of cancer, collected from polyclinics, and matched with the patient group in terms of sex and age. Patient sample collection was restricted to those diagnosed with colorectal cancer at the Kuwait Cancer Care Center during 2015–2020. Ethical approval was obtained from the local ethics committee of Kuwait University and the Ministry of Health Ethics Board following the guidelines set by the Declaration of Helsinki. All participants provided informed consent. Clinical data were collected via in-clinic interviews and a review of patient medical records when necessary. Their age, weight, height, and sex were recorded (Table 1). Of the 223 patient samples collected and of the 600 control samples collected, 48 and 171 were excluded from the study, respectively. The exclusion criteria included individuals who did not provide informed consent or blood samples at the time of the interview, those who were non-Kuwaitis, and those who had a condition that would interfere with our analysis or did not fit the final sex and age matching for our patient sample. Blood samples were collected in EDTA-coated tubes (Advance Medical Co., Riyadh, Saudi Arabia) and divided into two portions—one for enzyme-linked immunosorbent assay (ELISA) and the other for genomic DNA extraction.

### 2.2. Circulating IGF-1, IGFBP-2, and IGFBP-3 Measurement by ELISA

Plasma was separated using centrifugation of collected blood samples at 2000× *g* for 10 min at 4 °C and then aliquoted and stored at −20 °C. ELISA was performed using Human IGF-1, IGFBP-2, and 3 Quantikine ELISA kits (R&D Systems, Minneapolis, MN, USA), following the manufacturer’s protocol.

### 2.3. SNPs Genotyping

Total genomic DNA was extracted from blood samples using the Gentra^®^ Puregene^®^ DNA extraction Kit (Qiagen, Venlo, The Netherlands), as previously described [37]. DNA quantity and quality (260/280 ratio) were assessed using a NanoDrop spectrophotometer. The investigated SNP loci were selected from referenced publications. rs35767 is an A/G in the promoter region, and rs6214 is a C/T in the three prime untranslated region (3′-UTR) [38,39]. Samples were genotyped for both SNPs using Taqman^®^ Genotyping assays using previously published conditions (Life Technologies, Carlsbad, CA, USA; Applied Biosystems, Waltham, MA, USA) [37]. The allelic discrimination run was recorded using SDS v2.3 software, and alleles were called automatically or manually using a 7900HT Real-time PCR instrument (Life Technologies, Carlsbad, CA, USA; Applied Biosystems, Waltham, MA, USA).

### 2.4. Statistical Analysis

The Hardy–Weinberg equilibrium for each SNP was determined using an online calculator (https://gene-calc.pl/hardy-Weinberg-page, accessed 1 December 2021). Statistical analyses were performed using SPSS software (Version 28; SPSS Inc., Chicago, IL, USA) to compare the means between groups using analysis of variance (ANOVA). Additionally, Pearson’s chi-square test was used to assess the differences in genotype distribution between cases and controls. Patient and healthy control characteristics are expressed as the mean ± standard deviation (SD), and percentages were appropriate. Statistical significance was set at *p* < 0.05. Openepi (https://www.openepi.com/SampleSize/SSMean.htm, accessed 10 November 2023) was used for power calculations of IGFBP-2 levels and different tumor grades.

## 3. Results

### 3.1. Clinical Characteristics of Study Participants

In total, 175 patients with pathologically confirmed CRC and 429 matched controls were enrolled. The basic demographic and clinical characteristics of the study cohort are presented in Table 1. The mean age was 59.8 ± 11.9 for patients and 55.4 ± 11.3 for controls. The mean body mass index (BMI) range was 27.6 ± 7.3 for patients and 30.5 ± 7.1 for controls. All the patients underwent curative surgery and chemotherapy. The cancer stage and tumor differentiation grade were assigned whenever possible.

### 3.2. Plasma IGF-1, IGFBP-2, and IGFBP-3 in CRC Patients vs. Controls

Mean concentrations of IGF-1 circulating levels were lower in patients with CRC (123.1 ± 59.6 ng/mL) than they were in controls (213.6 ± 219.3 ng/mL) (*p* = 0.001), while mean concentrations of IGFBP-2 were higher in patients (1041.2 ± 756.8 ng/mL) than in controls (813.3 ± 528.0 ng/mL) (*p* = 0.002). Finally, IGFBP-3 levels were lower in patients (285.9 ± 121.6 ng/mL) than in controls (327.6 ± 180.2 ng/mL) (*p* = 0.014) (Table 2).

### 3.3. IGF-1 SNPs Are Not Associated with CRC or Circulating IGF-1 Levels

All investigated genotypic variants were observed in the study cohort. The allelic and genotypic frequencies of the two SNPs are summarized in Table 3. All tested alleles were in Hardy–Weinberg equilibrium.

Among these alleles, the most frequent wild-type allele expressed in both controls and patients was rs35767 G, whereas the most frequent variant was rs6214 T. There was no significant difference in the genotype and allele frequency of either SNPs between patients and controls. Specifically, no association between the genotype and CRC was observed.

### 3.4. Relationship between IGF-1 Protein Plasma Concentration Levels and rs6214 or rs35767

We compared the mean IGF-1, IGFBP-2, and IGFBP-3 protein levels between genotypes. No association was observed between the investigated SNPs and IGF-1 or IGFBP-2 plasma levels in patients and controls or in combination (Table 4).

However, comparing the IGFBP-3 plasma levels according to the rs6214 genotype in the patient and control groups separately yielded an interesting observation: in the patient group, IGFBP-3 levels increased with the C allele in a dose-dependent manner (*p* = 0.015), and this association was not observed in the control group (Table 5). Therefore, the C allele may be associated with increased IGFBP-3 levels in patients but not in controls.

### 3.5. Association of IGF-1, IGFBP-2, and IGFBP-3 Plasma Levels with Tumor Grade and Stage

Circulating levels of IGF-1, IGFBP-2, and IGFBP-3 were investigated for their association with tumor grade and stage. Only IGFBP-2 plasma levels were associated with tumor grade, and they increased with low grade and poor differentiation (Table 6, *p* = 0.026). No other associations were observed between any of the investigated proteins and tumor grade or stage.

## 4. Discussion

Differences in circulating IGF-1 and its inhibitors (IGFBP-2 and IGFBP-3) have been associated with the risk of several cancers, thus proposing these indicators as non-invasive biomarkers for cancer risk and prognosis [7,8,9]. In our study, plasma IGF-1 levels were lower in patients than they were in controls (Table 2). Although studies that previously investigated IGF-1 levels in CRC have yielded inconsistent results, some demonstrated that IGF-1 levels were higher in patients with cancer, whereas others reported that IGF-1 levels were lower in patients than they were in controls. However, studies that demonstrated that increased IGF-1 levels were associated with an increased risk of CRC were performed prior to the diagnosis of CRC, whereas those that revealed lower levels in patients compared to those in controls were performed in patients with advanced disease, after the beginning of treatment and disease progression. Therefore, it appears that higher levels of IGF-1 were detected in patients before diagnosis up to disease onset; however, after disease onset, IGF-1 has been demonstrated to decrease with disease progression [41]. This has been observed in a number of cancers, including breast cancer, hepatocellular carcinoma, and CRC, and is in agreement with our findings [42,43,44].

In our study cohort, IGFBP-2 levels were higher in patients with CRC than they were in the controls (Table 2), and IGFBP-2 levels increased with increasing histological grade of tumor differentiation (Table 6). As IGFBP-2 is the main binding protein and inhibitor of IGF-1 and therefore inhibits its pro-proliferative action, it would be logical for IGFBP-2 to be downregulated in patients with cancer. Conversely, high IGFBP-2 expression has been reported in the serum and tissues of several cancers [44]. In addition to its role as an IGF-1 inhibitor, IGFBP-2 is a developmental protein that is highly expressed during fetal development and embryogenesis; however, its expression decreases significantly after birth [45]. Additionally, IGFBP-2 is upregulated in several cancers and promotes several pathways involved in oncogenic signaling, including the stimulation of proliferation, epithelial–mesenchymal transition, invasion, metastasis, and angiogenesis, all of which are independent of IGF-1 [46,47]. Furthermore, IGFBP-2 levels were positively correlated with tumor size and decreased significantly in patients following curative surgery [48]. Finally, IGFBP-2 levels were low in well-differentiated tumors and normal tissues but high in poorly differentiated tumors [49]. This suggests that IGFBP-2 circulating levels are positively associated with tumor load and can be used to measure disease progression and response to therapy. Therefore, they can be used as prognostic biomarkers.

In contrast to IGFBP-2, IGFBP-3 expression was lower in patients than it was in controls (Table 2) and was not associated with tumor grade or stage (Table 6). Again, this is consistent with previous reports of an IGFBP-2 correlation with an increased malignant status of the tumor but not IGFBP-3. This correlation was reported in cancers of the colon, lung, ovaries, prostate, and central nervous system [50,51,52,53,54]. The most well-known function of IGFBP-3 is IGF-1 inhibition. IGFBP-3 also increases IGF-1 function by stabilizing IGF-1 and protecting it from degradation. High circulating concentrations of IGFBP-3 are associated with reduced cancer risk; however, once cancer develops, IGFBP-3 levels in cancer patients drop significantly compared to levels in control groups [55,56,57]. Decreased IGFBP-3 expression correlates with disease progression and exhibits antitumor activities that are IGF-1-independent, including pro-apoptotic and anti-proliferative functions [58,59,60]. Additionally, IGFBP-3 has been demonstrated to induce apoptosis and decreases survival when stimulated by p53 in response to DNA damage in breast cancer and CRC cells [61]. Taken together, these results lead to the conclusion that IGFBP-3 exhibits tumor suppressor activity; therefore, it would be logical for IGFBP-3 to be reduced in patients with cancer. It remains unclear if this is the cause or result of cancer.

Most CRC cases are sporadic and may be caused by a plethora of lifestyle and environmental factors [62]. However, approximately 30% are inherited. Nearly 5% are associated with highly penetrant inherited mutations, whereas the remaining 25% are likely to be caused by variations in less penetrant but more common single genes, including SNPs [63].

Our study revealed no association between IGF-1 SNPs and CRC (Table 3). All genotypes for both SNPs were represented in the cohort and in Hardy–Weinberg equilibrium. The SNPs rs6214 and rs35767 have been investigated for their association with different cancers, but the results have been inconsistent. While certain studies demonstrated an association, others failed. Interestingly, studies that observed an association reported that it may be race specific [39,64].

The rs6214 SNP is located in the 3′-UTR, and this may indicate its importance in the translation, stability, and localization of IGF-1 mRNA. rs35767 is located in the promoter region, and this may indicate that it affects IGF-1 mRNA expression, and therefore, protein levels [65]. Both SNPs possess the potential to affect circulating IGF-1 levels; however, inconsistent results have been obtained regarding the association between these SNPs and circulating IGF-1 levels by many studies [35,39]. One study reported that this association may be race specific, as it was present in Caucasian women but absent in African American women [66]. In our study, none of the SNPs were associated with CRC or IGF-1 and IGFBP-2 levels (Table 3 and Table 4). However, it is interesting to note that rs6214 was associated with IGFBP-3 levels in patients but not in controls, where the C allele was associated with an increase in IGFBP-3 expression (Table 5). This may indicate that this SNP affects IGFBP-3 expression via IGF-1, as it has been reported that IGF-1 increases IGFBP-2 and -3 expression in a cell-dependent manner [67,68]. Although rs6214 is in the UTR of exon 4, it may have regulatory functions or may be in strong linkage disequilibrium with functional variants that influence IGF-1 [69]. Furthermore, as IGFBP-3 expression is affected by IGF-1, it may also be affected. These findings warrant further investigation.

Our study possesses some limitations. First, the sample size was moderate for this type of study, and this limited its statistical power. Also, a limitation to overcome is increasing the number of patients with tumor grade information to detect a power above that of 70%. Additionally, this was a retrospective clinical study. Finally, the lack of information on CRC risk factors for most patients such as diet, physical activity, and smoking limited our ability to evaluate the association between these factors, IGF-1 levels, and CRC risk.

## 5. Conclusions

In summary, we investigated the circulating levels of IGF-1 and its binding proteins IGFBP-2 and IGFBP-3 in patients with CRC and their association with stage and grade. All three proteins exhibited different expression levels in patients and controls. IGF-1 and IGFBP-3 levels were higher in controls than they were in patients, whereas IGFBP-2 levels were higher in patients than they were in controls. Only IGFBP-2 was associated with an increased tumor grade. We also examined if SNPs in IGF-1 were associated with cancer risk, IGF-1, IGFBP-2, and IGFBP-3 levels, and/or both and observed no association. We believe that these proteins can be useful as early detection and prognostic biomarkers in CRC and other cancers; however, additional studies investigating other SNPs at these gene loci in different populations and ethnicities are required to confirm this hypothesis.

## Figures and Tables

**Table 1:** Demographics and clinical features of the study cohorts.

Characteristics of the Study Cohorts	CRC Patients (*n* = 175)	Controls (*n* = 429)
Male, *n* (%)	80 (45.7)	226 (52.7)
Age	59.8 ± 11.9	55.4 ± 11.3
18–24	0	5
25–34	1	11
35–44	19	48
45–54	45	138
55–64	40	131
65–74	51	68
>75	20	28
Age of onset	58.17 ± 12.6	-
BMI	27.6 ± 7.3	30.5 ± 7.1
Stage		
I	2	
II	17	
III	34	
IV	32	
Undetermined	90	
Grade		
Low	27	
Intermediate	76	
High	16	
Undetermined	56	

Values provided are the mean ± standard deviation or number of patients (*n*) (%). Body mass index (BMI), colorectal cancer (CRC).

**Table 2:** IGF-1, IGFBP-2, and IGFBP-3 plasma levels in CRC patients and controls.

Plasma Protein (ng/mL)	Patients (*n* = 167)	Controls (*n* = 159)	*p* Value
IGF-1	123.1 ± 59.6	213.6 ± 219.3	**0.001**
IGFBP-2	1041.2 ± 756.8	813.3 ± 528.0	**0.002**
IGFBP-3	285.9 ± 121.6	327.6 ± 180.2	**0.014**

Protein levels are presented as mean ng/mL ± standard deviation. Bold indicates statistical significance.

**Table 3:** Association of the IGF-1 SNPs rs35767 and rs6214 with CRC.

IGF-1 SNP	Patients *n* (%)	Allele Frequency	Control *n* (%)	Allele Frequency	*p*
rs35767					
GG	109 (62.3%)	G = 0.78	269 (62.7%)	G = 0.72	0.946
AG	56 (32%)	A = 0.22	133 (31%)	A = 0.22	
AA	10 (5.7%)		27 (6.3%)		
rs6214					
CC	67 (38.2%)	C = 0.63	204 (47.55%)	C = 0.68	0.114
CT	85 (48.65%)	T = 0.37	179 (41.72%)	T = 0.32	
TT	23 (13.14%)		46 (10.7%)		

Number of patients (*n*) (%).

**Table 4:** Comparing the means between the IGF-1 genotypes and IGF-1 plasma levels and the IGF-1 SNPs to IGF-1 levels in the study cohort.

SNP-Genotype	IGF-1 Total Study Cohort	*p*	Patients IGF-1	*p*	Control IGF-1	*p*
rs6214 genotype						
CC	172.9 ± 160.4		126.7 ± 55.5		209.8 ± 202.5	
CT	153.4 ± 136.8		120.8 ± 63.6		194.5 ± 185.8	
TT	199.2 ± 259.1	0.276	120.5 ± 57.8	0.822	314.7 ± 379.0	0.158
rs35767 genotype						
GG	174.8 ± 189.6		125.1 ± 60.3		230.5 ± 258.2	
AG	155.4 ± 127.1		121.7 ± 60.6		189.0 ± 163.2	
AA	160.4 ± 87.6	0.276	110.1 ± 47.7	0.736	199.2 ± 92.3	0.529

Protein levels are presented as mean ng/mL ± standard deviation.

**Table 5:** Analysis of the association of rs6214 with IGFBP-3 levels in the study cohort.

rs6214 Genotype	IGFBP-3 Total Study Cohort	*p*	Patients IGFBP-3	*p*	Controls IGFBP-3	*p*
CC	324.2 ± 154.9		316.0 ± 130.9		330.8 ± 172.2	
CT	297.4 ± 154.3		275.8 ± 115.2		325.1 ± 190.8	
TT	270.3 ± 144.8	0.108	235.6 ± 96.5	**0.015**	321.3 ± 187.9	0.972

IGFBP-3 levels are expressed as mean ng/mL ± standard deviation. Bold indicates statistical significance.

**Table 6:** Association of IGF-1, IGFBP-2, and IGFBP-3 plasma levels with tumor grade and stage.

Tumor Grade	IGF-1	*p*	IGFBP-2	*p*	IGFBP-3	*p*
Low	137.9 ± 78.2		610.6 ± 403.5		302.7 ± 95.7	
Intermediate	117.9 ± 54.3		1021.3 ± 764.1		279.3 ± 134.4	
High	125.1 ± 61.4	0.368	1108.8 ± 821.4	**0.026**	310.2 ± 135.6	0.554
Tumor stage						
1	143.3 ± 29.2		782.7 ± 765.1		414.3 ± 4.2	
2	118.7 ± 56.0		1079.1 ± 982.2		274.6 ± 131.1	
3	132.9 ± 67.2		863.7 ± 763.4		286.6 ± 86.7	
4	115.7 ± 77.2	0.750	1054.6 ± 808.1	0.749	291.6 ± 137.9	0.470

Protein levels are expressed as mean ng/mL ± standard deviation. Bold indicates statistical significance. Tumor grade: low, well differentiated; intermediate, moderately differentiated; high, poorly differentiated. Staging was performed according to American Joint Committee on Cancer Guidelines [40].

## Data Availability

The datasets supporting the conclusions of this study are included in the Supplementary File.

## Supplementary Materials

The following supporting information can be downloaded at: https://www.mdpi.com/article/10.3390/biomedicines11123166/s1, Table S1: Genotypic and clinical data for patients and controls of the study.
